# The 3rd Antibody Validation meeting: Bath UK 20-21 ^st^ September 2018

**DOI:** 10.12688/f1000research.17645.1

**Published:** 2018-12-31

**Authors:** Simon L. Goodman

**Affiliations:** 1 Independent Freelance Writer, Darmstadt, Hessen, Germany

**Keywords:** Meeting report, antibody, validation

## Abstract

The International Antibody Validation meetings offer a welcome British forum for discussing this important topic, which is existentially crucial for the biological sciences community. Now in its 6th year, the biennial meeting is organized by Andrew Chalmers (University of Bath; CiteAb), this year with Carly Dix (Astra Zeneca).  The organizers gathered some 100 members of industry and academia, producers and users, for a day and a half to describe their efforts to ensure that their antibodies have the desired specificity and selectively for well-defined molecular targets. The meeting is largely available as WebCasts ( http://www.antibodyvalidation.co.uk/past-events/2018).

You might say things are somewhat Grimm. “Communities were overrun by tiny creatures. The friendly ogre, HUGO, had shown that there were some 20,000 of them in each “town”, in all shapes sizes, lengths and breadths. Chewing, modifying and moving things, and making more and more of themselves. But this all went on rather unpredictably, for the community elders were a bit slow and couldn’t even see, find or count most of the creatures, let alone discover where they dwelt. They were growing desperate until, one day, some gaily clad fellows, call them Catologschen, arrived at their desks, promising to solve their every problem; against suitable remuneration of course. They, and they alone could find, identify, enumerate and gather the creatures and lead them off to somewhere cosy, and undoubtedly fit for publication. Oh joy! But the community rashly refused to pay the not inconsiderable validatory price, so Catalogschen whistled together all the children, both doctoral and postdoctoral, and led them to the Wicked Witch who makes those endlessly useless and retracted publications, and offers nostrums targeting mythical molecules”.

Thus, in short, the antibody validation problem. You may dislike whimsey, but certainly you dislike useless antibodies far more ^1, 2^. The last decade has seen journal editors ^3^, users both peeved ^4, 5^ and astounded ^6– 9^, and influential opinion leaders ^10– 12^ trying to awaken the research community to the issues that inadequately validated research tool antibodies create for scientific research. Hence the necessity for meetings like this.

Mathias Uhlen (KTH Sweden) kicked off the meeting and suggested that there are no “bad antibodies” only inadequate validations. His team in Stockholm have driven major efforts which have led to the Human Protein, Cell, and Pathology Atlases ^7^. They have developed some 55000 in-house reagents, a number expanding at some 7000 antibodies per year.

Jason Li (Proteintech, USA) had a less benign viewpoint. Reviewing two Chinese cities, he discovered some 74 antibody supply companies and he speculated that, the real estate bubble in danger of bursting, this was where money might be flowing. Apparently not all validate their antibodies effectively. In an entertaining talk, Jason reminded the meeting that the specificity of antibody binding is dependent on concentration; higher concentration permits binding to lower affinity epitopes. He emphasized the need for perfect and appropriately glycosylated antigens for immunization and validation, and that titration of reagents is crucial to optimize signal-to-noise in the particular experimental context in use.

There was discussion over appropriate immunogens. Some companies favour using polypeptides, but Birte Aggeler (Bio-Techne/R&D systems; USA) emphasized that native and biologically active proteins are excellent immunogens. She noted the need to constantly re-evaluate existing antibodies as knowledge increases. Rigorous quality control (QC) and production strategies were crucial for reproducibility, as emphasized by the ISO9001 and ISO13485 compliance of Bio-Techne production–some 13000 antibodies having been made on site.

Alejandra Solache (Abcam, UK) also emphasized the need for rigorous QC and ISO compliance. Since 2015 Abcam moved to antibody validation at scale using CRISPR-Cas9 target knock-out cell lines. Such knock-out cell lines are increasingly commercially available, and specific loss of signal following knock-out is a robust way of demonstrating antibody specificity for a target in many assay formats ^13^.

Roberto Polakiewicz (Cell Signalling Technology [CST], USA) described the strong research culture of CST, which encourages routine rigorous validation QC in characterizing in-house antibodies against signalling pathways. He noted that success rates for producing well validated antibodies were low, and emphasized that users should strictly follow the defined permeabilization and fixation protocols for each reagent. A point often lost to users. CST still supports “high quality” polyclonal antibodies, because of the difficulties of raising robust monoclonal reagents against signalling-pathway modifications, but they are shifting toward a rabbit recombinant monoclonal society ^14^.

Deepa Shankar (Thermo Scientific, USA) showed the value of CRISPR-Cas9 knockdowns in antibody validation, and discussed linking antibody validation to down-stream signalling pathways, with the Nfkb pathway as one of many examples. Thermo constantly clean their catalogue of inappropriately non-fit-for-purpose reagents. Antibodies can be further validated by linking their reactivity to the state of pathway activation. She discussed efforts to specify epigenetic targets using the SNAP-ChiP nucleosome barcoding techniques with recombinant and modified nucleosomes ^15^.

From the user side Richard Goodwin (AstraZeneca, UK) described his teams’ efforts using imaging mass spectrometry (Fluidigm system) for immunohistochemistry (IHC), correlated to multimodal mass spectrometry, to investigate drug distribution and metabolism. Fluidigm rare-earth isotope antibody labelling allows ~ 50-plex antibody staining without the spectral-overlap issues of high-multiplex fluorescence techniques. He amusingly noted that they needed to move to their own (i.e. adequately) validated antibodies after attempting to use those from Fluidigm.

Naturally, the higher the multiplexing, the greater the possibility of steric interference becomes, as emphasized by Gemma Jones (Astra Zeneca, UK). Even in low multiplex immunohistochemistry (IHC) she noted that it was crucial to individually establish the specificity of each primary antibody, as well as of the combination. Unexpected perturbation of target sites often occurs. This is especially problematic where quantitative image analysis of staining profiles is being used for clinical diagnosis. It is risky to establish a multiplex assay where the staining intensity of one partner is modulated by the reagents used to stain another.

Jan Roger (GSK, UK) discussed antibody-based target validation for drug discovery. A genomics selection cascade filtered targets to 10-100 candidates, and then IHC was used for target localization, while the “Trim-away” ubiquitin ligase technology was used ^16^ for intracellular target validation, independent of protein life time or RNA expression level. He emphasized that the targets of interest were unknown-unknowns, with no pre-validated antibodies. This is not unusual in the pharmaceutical industry, and requires a more than usually extensive validation effort, including correspondence between Western blot and protein array labelling, and the use of independent biological triplicate organ samples of diseased and control tissues in IHC validation efforts. He emphasized that the highest quality of target-tissue, of definitive positive and negative controls, and optimized probes are all essential for robust validation of drug targets. GSK strategy is to publish all antibody validation data.

Dagmar Ehrnhoefer (BioMedX; Germany) described their team’s validation effort on commercial antibodies against the diverse post-translational modifications (PTMs) of mictrotubule-binding protein Tau – aiming to find correlation with neurodegenerative diseases. This is hindered by the extreme diversity of Tau PTMs. Focusing on the ~70 potential phosphorylation sites, the team found that while many (25%) of the commercial “site specific” reagents were non-specific, the remainder could be well validated by phospho-peptide and Western blotting, and by IHC.

In another example, Peter Kloehn (MRC Prion Unit) discussed the potential of systematically finding therapeutic antibodies with enhanced therapeutic index by differential antibody selection of non-binders on cancer cells, which bound cryptic epitopes exposed when the cancer cell surface proteins were denatured.

There were two contributions from mass spectroscopists, one from Fridtjof Lund-Johansen (Oslo University Hospital, Norway), who described his MS platform for proteomic antibody validation. PAGE separation of protein mixtures enables size fractionated clusters to be analysed by IP-MS, shotgun-MS and parallel antibody arrays. This impressive technology is a valuable addition to antibody validation.

A second, from Kathryn Lilley (Uni. Cambridge; UK), described her teams’ efforts to discover how the proteome gets to correct positions in the cell using the LOPIT isotope (antibody-independent) labelling. Mass spectrometry of biochemical density gradient fractions allows hundreds of proteins to be mapped to compartments and even to large protein complexes.

Two discussion forums addressed what suppliers and end-users can do to improve antibody validation. In principle no new concepts emerged from these open talks – but it is becoming clear that at least those suppliers represented in Bath are taking their responsibilities seriously, and the users recognize that the reagents that they have in their laboratories must be validated as fit-for-purpose.

The meeting productively brought together producers and users, and was flavoured by the talks of quality producers describing their increasingly extensive validation efforts. It would perhaps would have benefited from the presence of more academic contributions of validation problems they have wrestled with – but clearly in such a short meeting (1.5 days) an appropriate balance is hard to achieve. There can certainly not be enough of such efforts to highlight the issues involved, and to help the community optimize its effective use of antibodies in research. The meeting is largely available as WebCasts ( http://www.antibodyvalidation.co.uk/past-events/2018).

## Data availability

No data are associated with this article.
